# Exploring Novel Umami Peptides from Bovine Bone Soups Using Nano-HPLC-MS/MS and Molecular Docking

**DOI:** 10.3390/foods13182870

**Published:** 2024-09-10

**Authors:** Zheng Yang, Wanying Li, Ran Yang, Lingbo Qu, Chunxiang Piao, Baide Mu, Xiaodi Niu, Guanhao Li, Changcheng Zhao

**Affiliations:** 1 School of Life Science, Zhengzhou University, Zhengzhou 450001, China; 15737372369@163.com (Z.Y.); lwyzzu123@163.com (W.L.); 2 Food Laboratory of Zhongyuan, Zhengzhou University, Zhengzhou 450001, China; yangran@zzu.edu.cn (R.Y.); qulingbo@zzu.edu.cn (L.Q.); 3 College of Chemistry, Zhengzhou University, Zhengzhou 450001, China; 4 Agricultural College of Yanbian University, Yanji 133000, China; cxpiao@ybu.edu.cn (C.P.); mubaide@ybu.edu.cn (B.M.); 5 Engineering Research Center of North-East Cold Region Beef Cattle Science & Technology Innovation, Ministry of Education, Key Innovation Laboratory for Deep and Intensive Processing of Yanbian High Quality Beef, Ministry of Agriculture and Rural Affairs, College of Agricultural, Yanbian University, Yanji 133000, China; 6 College of Food Science and Engineering, Jilin University, Changchun 130062, China; niuxd@jlu.edu.cn

**Keywords:** bovine bone, umami peptides, molecular docking, T1R1/T1R3

## Abstract

In this study, umami peptides were screened and characterized from bovine bone soups manufactured via atmospheric and high-pressure boiling. Peptide fractions with molecular weights less than 3 kDa were selected for peptide sequencing using LC-MS/MS, the toxicity prediction of the umami peptides was carried out by using an website, and the peptides were screened according to the binding energy, i.e., three peptides including YDAELS, TDVAHR, and ELELQ were selected. The three umami peptides were further synthesized, and their umami thresholds were determined through sensory evaluation and electronic tongue analysis, ranging from 0.375 to 0.75 mg/mL. All three peptides exhibited a significant synergistic taste enhancement effect when combined with MSG (monosodium glutamate) solution. The molecular docking of the umami peptides with the T1R1/T1R3 receptor revealed the mechanism of umami presentation, and the main interaction forces between the three umami peptides and the receptor were hydrogen bonding, electrostatic interactions, and hydrophobic interactions.

## 1. Introduction

Bovine bones, constituting approximately 10% of a cattle’s total weight, are a significant by-product of the meat industry [1]. According to 2023 data from the National Bureau of Statistics of the People’s Republic of China, approximately 31.57 million cattle were slaughtered, which reflects an increase of 4.88% compared to the previous year. These bones are rich in protein, organic acids, and other nutrients, with protein accounting for about 11% of their composition [2]. The extraction and utilization of these nutrients from bovine bones are crucial for maximizing their economic potential. Notably, bovine bone soup serves as a nutrient-rich medium, abundant in protein, fat, bone collagen, adhesion proteins, cells, marrow, and other nutrients, making it an effective medium for extracting peptides [3].

Umami, recognized as one of the five fundamental tastes, is derived from free amino acids, nucleotides, peptides, organic acids, and their derivatives, which interact with specific receptors [4]. Recent concerns over synthetic umami agents have spurred research into bioactive peptides as natural alternatives [5]. Studies have identified small molecular peptides with umami properties in bovine bone soup [6], yet the specific composition and physiological characteristics of these peptides remain unclear. Research has elucidated that umami receptors are primarily G protein-coupled receptors, notably the heterodimeric taste receptor type 1 members 1/3(T1R1/T1R3) and metabotropic glutamate receptors mGluR1 and mGluR4 [2]. According to previous studies, a total of eight umami receptors have been identified to date. Among them, the heterodimer T1R1/T1R3 is widely acknowledged as a primary contributor to umami taste perception. This assertion is supported by the interaction capabilities of the extracellular Venus Flytrap (VFT) domain within the T1R1/T1R3 heterodimer, which may bind orthogonists and subsequently enhance umami flavor detection synergistically. Further validation of this role is provided by structural features including the cysteine-rich domain (CRD), the seven-span transmembrane region (7TM), and the cytoplasmic domains of the T1R subunits [7]. Given the significant implications of these findings, considerable efforts have been directed toward using macromolecular simulation and sensory studies to explore the mechanisms of umami perception and to delineate the molecular interactions between umami peptides and the T1R1/T1R3 heterodimer. In addition, the effectiveness of umami peptides as signaling molecules hinges on their structural features, including positively charged, negatively charged, and hydrophobic groups, which must align with specific receptor sites or amino acid residues [8]. Beyond peptides, bovine bone soup also contains significant collagen proteins, whose structure is influenced by heating methods and intensities [1].

Recognizing the profound impact of peptide composition on functionality, this study aims to investigate new umami peptides from bovine bone soup prepared using conventional boiling and high-pressure boiling methods, and clarify the mechanism of its taste. LC-MS/MS and molecular docking techniques were used to identify and screen the umami peptides. In addition, the taste characteristics and threshold of the screened peptides were determined through sensory evaluation and electronic tongue analysis. Finally, through molecular docking with T1R1/T1R3, the umami mechanism of these peptides was deeply understood, and the umami peptide library was enriched.

## 2. Materials and Methods

### 2.1. Materials and Reagents

Bovine bones (Miyang Xia Nan cattle) were purchased from Zhengzhou wholesale market (Zhengzhou, China). DL-dithiothreitol (DTT, AR), iodoacetamide (IAM, AR), formic acid (FA, LC-MS grade), acetonitrile (CAN, LC-MS grade), and ammonium bicarbonate (NH_4_HCO_3_, LC-MS grade) were purchased from Sigma (St. Louis, MO, USA).

### 2.2. Sample Preparation

The samples were prepared according to the following protocol: (1) two bovine bones of similar quality were selected, thawed, and soaked in pure water for 4 h to remove blood water, remove the meat on the surface of the bovine bone and the internal bone marrow, and then soaked for 2 h. (2) The soup was cooked using atmospheric pressure boiling (BBS) and high-pressure boiling (HBBS), respectively. The mass ratio of cow bone to water was 1:3, and the proportion of water was not higher than the proportion of cow bone. Atmospheric cooking using an induction cooker (D812H, Zhejiang Supor Co., Ltd., Hangzhou, China) was used to cook the soup for 2 h, while high-pressure cooking was conducted in a vertical-pressure steam sterilizer (YXQ-100G, Shanghai Boxun Medical Biological Co., Ltd., Shanghai, China) for 2 h [2]. (3) The bone soups were cooled to room temperature and then the floating foam on the surface of the soup was removed and cleaned. After that, the boiled liquid was collected in a conical bottle and filtered with gauze to remove impurities. (4) The bovine bone soup was centrifuged using a high-speed refrigerated centrifuge (KH23A, Kaida Scientific Instrument Co., Ltd., Changsha, China) at 8000 r/min for 10 min at 4 °C. After centrifuging, the clear liquid was collected. (5) The ultrafiltered fractions were carefully dispensed into EP tubes for centrifuging at 4000 rpm for 15 min. Upon completion of centrifugation, constituents from both the outer and inner compartments of the ultrafiltration tube were separately collected. Notably, the molecular weight of the materials collected from the outer compartment was below 3 kDa [9]. Subsequently, these ultrafiltered components were stored at −20 °C in a refrigerator.

### 2.3. LC-MS/MS-Based Peptide Sequencing of Peptide Extract from Bovine Bone Soups

The characterization of umami peptides derived from HBBS and BBS was conducted using a Nano-HPLC-MS/MS system (Thermo Fisher Scientific, Waltham, MA, USA). Approximately 3 μL of the peptide sample was injected onto an analytical column (Reprosil-Pur 120 C18-AQ 3 μm, 100 μm i.d. × 180 mm) and subjected to separation via a 60 min linear gradient, transitioning from 2% B (A: 0.1% formic acid aqueous solution; B: 80% ACN/0.1% formic acid aqueous solution) to 100% B at a flow rate of 600 nL/min and a temperature of 40 °C [10]. The mass spectrometer operated in data-dependent acquisition mode, automatically alternating between MS and MS/MS. Full-scan MS spectra (m/z 200–1800) were captured with a mass resolution of 70,000. The raw mass spectrometry data were processed for peptide sequencing using PEAKS Studio 10.6 De novo software, with the following parameters: fixed modification: carbamidomethyl (C); variable modification: oxidation (M) and acetyl (N-term); enzyme: none; missing sites: 3; error in primary mass spectrometry: 20 ppm; error in secondary mass spectrometry: 0.02 Da. The mass spectrometry-generated raw files were analyzed using PEAKS Studio 10.6 De novo to generate a comprehensive list of peptides.

### 2.4. Screening of Umami Peptides

Peptides extracted from bovine bone soups were predicted for toxicity using an online prediction method (https://webs.iiitd.edu.in/raghava/toxinpred/multi_submit.php (accessed on 15 April 2024), and peptides that might be toxic were removed. We then selected a Denovo score greater than 95 peptides for subsequent screening. This is because in LC-MS/MS analysis, the higher the Denovo score, the higher the peptide score, which means the higher the probability that the peptide is contained in the fraction, and the peptide has a higher overall score. The peptides containing D or E residues in the amino acid sequence were selected due to umami peptides sequences usually contain glutamate, aspartate, glutamate and aspartate can bind to human umami receptors and play a role in the activity of umami receptors. From the above peptides, we selected peptides with amino acid sequence lengths of 4 to 6. Because the sequence length of umami peptides is usually 3 to 9 amino acids, when the sequence length is 4 to 6 amino acids, it has a stronger umami. Subsequently, a molecular docking simulation was conducted to evaluate the interaction between the selected peptides and the umami receptor T1R1/T1R3. The peptides that could not be successfully docked were deleted. After docking, peptides with lower docking binding energies were ranked according to their docking binding energies, and peptides with lower docking binding energies had a higher direct affinity for the receptor, so peptides with low docking energies were selected.

### 2.5. Molecular Docking

To obtain the 3D structure of the major umami receptor T1R1/T1R3, homology modeling techniques were utilized. The amino acid sequence composition of T1R1 and T1R3 was downloaded from the UniProtKB database (https://www.uniprot.org/ (accessed on 15 April 2024)) [11], and mGluR1 (PDB ID: 1EWK) from the RCSB database (http://www.rcsb.org/ (accessed on 15 April 2024)) was selected as the homology modeling template [12]. The homology model of the composite sequence containing T1R1 and T1R3 was constructed online using the SWISS-MODEL server (SWISS-MODEL(easy.org)) website, and the preliminary homology model was imported into Discovery Studio 2019, and the model was optimized using the minimization method. After that, the model was imported into SAVES v6.0 (SAVESv6.0—Structure Validation Server (ucla.edu)) to verify the reasonableness of the model [13]. Finally, the model with the proportion of amino acids in the Ramachandran plot that are in a reasonable amino acid region was identified as the model for subsequent molecular docking. The energy of the peptides was minimized, and the minimized 3D model was confirmed to be the peptide model for the subsequent molecular docking. Ligand small molecules and receptor macromolecules were processed before docking, after which molecular docking of the umami peptide and T1R1/T1R3 homology model was performed using CDOCKER in Discovery Studio 2019 Client.

### 2.6. Solid-Phase Synthesis of Umami Peptides

The screened umami peptides from the bovine bone soups were synthesized by Hongtai Biotechnology Co., Ltd. (Shanghai, China) using the solid-state synthesis method with purity no lower than 95%.

### 2.7. Sensory Evaluation

The sensory analyses were performed according to previous research based on selecting four individuals with a background in food research from the School of Life Sciences at Zhengzhou University and randomly identifying three additional students with good taste discrimination skills [14]. In this study, individuals participating in the sensory experiment were informed of the protocol and volunteered to participate. Prior to evaluation, all participants were requested to provide informed consent. All participants’ rights and privacy were well protected, and consent was given for the collection and use of their personal information and related experimental data (ethics approval code: ZZUIRB2024149). Sensory evaluations of three 1 mg/mL umami peptide solutions were performed at room temperature for acidity, sweetness, bitterness, umami, saltiness, and astringency [15].

Three synthetic peptides (10 mg) were dissolved in 10 mL of ultrapure water and the pH was adjusted near to 6 using NaHCO_3_. Since the taste of the umami peptide solution was judged to be very mild during pre-tasting, the panelists were required to determine the taste characteristics, including sour, sweet, bitter, salty, umami, and astringent. Umami thresholds for synthetic peptides were analyzed using triangular taste tests. Briefly, we employed synthetic peptides gradually diluted with ultrapure water at a 1:1 ratio (*v*/*v*) using a triangulation test to analyze samples at each dilution concentration (from high to low), which included one experimental group and two blank groups. When the panelists could not distinguish the experimental group from the three sets of solutions, the average of the concentrations at that level and the concentration of the previous gradient were recorded as the threshold value for the sample. Umami enhancement threshold experiments, on the other hand, involved dissolving the synthetic peptide in 0.35% MSG (*w*/*v*) solution, and the blank control group was replaced with 0.35% MSG (*w*/*v*) solution. All other conditions were kept constant and thresholds were analyzed using the same method.

### 2.8. Electronic Tongue Analysis

The electronic tongue probe was pre-conditioned for 24 h. The electrodes were then immersed in three synthetic peptide solutions for a minimum of 24 h before being engaged with the nine sensors (intensity, aftertaste—A, aftertaste—B, sourness, saltiness, bitterness, astringency, sweetness, and umami sensors) integrated within the electronic tongue system (Alpha MOS Co., Toulouse, France) [16]. Prior to use, the taste sensors were immersed in a reference solution composed of 30 mM KCl and 0.3 mM tartaric acid, while the electrodes were placed in a 3.3 mM KCl solution. The sensors underwent calibration for about 30 min to ensure accurate measurements. Each sample was subjected to measurement three times under ambient temperature conditions.

### 2.9. Statistical Analysis

Statistical analysis was conducted using SPSS software (version 19.0, SPSS Inc., Chicago, IL, USA). Data are presented as the means ± standard deviations (SDs). A radar chart was generated using Origin 2018 (Origin Lab Corporation, Northampton, MA, USA). Significant differences were assessed using one-way analysis of variance followed by Duncan’s multiple range test, with *p* < 0.05 considered statistically significant.

## 3. Results and Discussion

### 3.1. Screening Results and Determination of Umami Peptides

**Figure 1:**
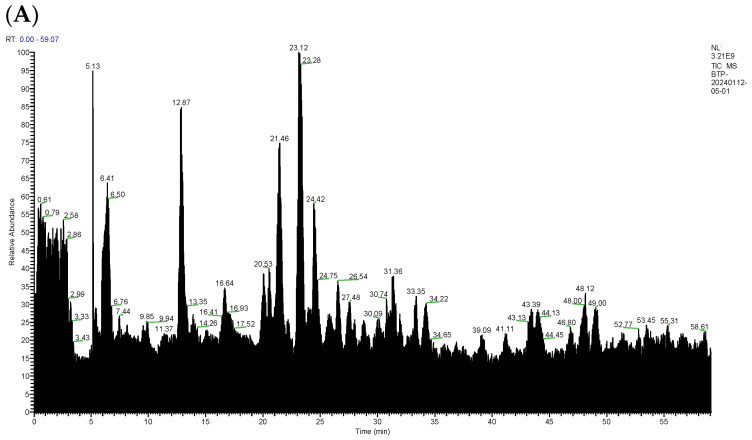

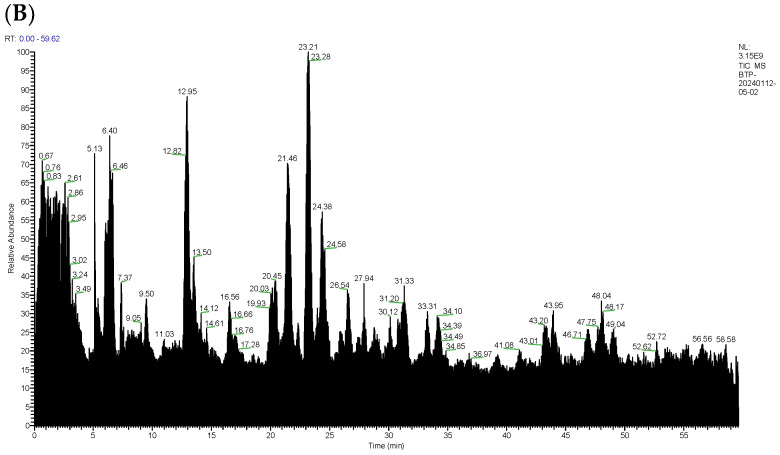
Total ion flow chromatogram of cured peptides of HBBS (**A**) and BBS (**B**).

Figure 1 shows the total ion flow chromatograms of samples HBBS and BBS, which identified a total of 656 peptides in HBBS and 456 peptides in BBS, for a total of 1112 peptides. The top ten peptides are shown in Table 1, and all peptides were screened for subsequent analysis [17]. The toxicity of the 1112 peptides was predicted online, and there were 7 toxic peptides in HBBS and 40 peptides in BBS. After deleting these toxic peptides, 649 peptides remained in HBBS and 416 peptides remained in BBS. Then, the peptides with a Denovo score greater than 95 were selected, 11 peptides with HBBS with a Denovo score greater than 95, and 12 peptides with BBS with a Denovo score greater than 95, leaving a total of 23 peptides. Further screening was based on the fact that umami peptides often contain glutamic acid and aspartic acid and their length. Therefore, peptides containing glutamic acid or aspartic acid and with a peptide length of four to six amino acids were selected for further analysis [18]. There were eight remaining in HBBS and ten remaining in BBS. Peptides with repetitive sequences were deleted, leaving a total of 16 peptides.

Finally, molecular docking screening was performed to construct three-dimensional models of these 16 peptides and molecular docking screening was performed with T1R1/T1R3. The binding energies of the docked molecules were ranked in ascending order, and the docking binding energies were shown in Table 2. According to the ranking of the docking binding energies, the peptides with the top ranking (i.e., smaller docking binding energies) were selected for analysis, and the three peptides, namely, YDAELS, TDVAHR, and ELELQ, were finally selected for further analysis [19]. The mass spectral information of these three peptides is displayed in Table 3, which includes sequence number, confidence level, chain length, charge-to-mass ratio, charge number, liquid chromatographic retention time, relative intensity, molecular weight, and detected difference between theory and molecular weight.

### 3.2. Construction of the T1R1/T1R3 Model of the Umami Receptor

**Figure 2:**
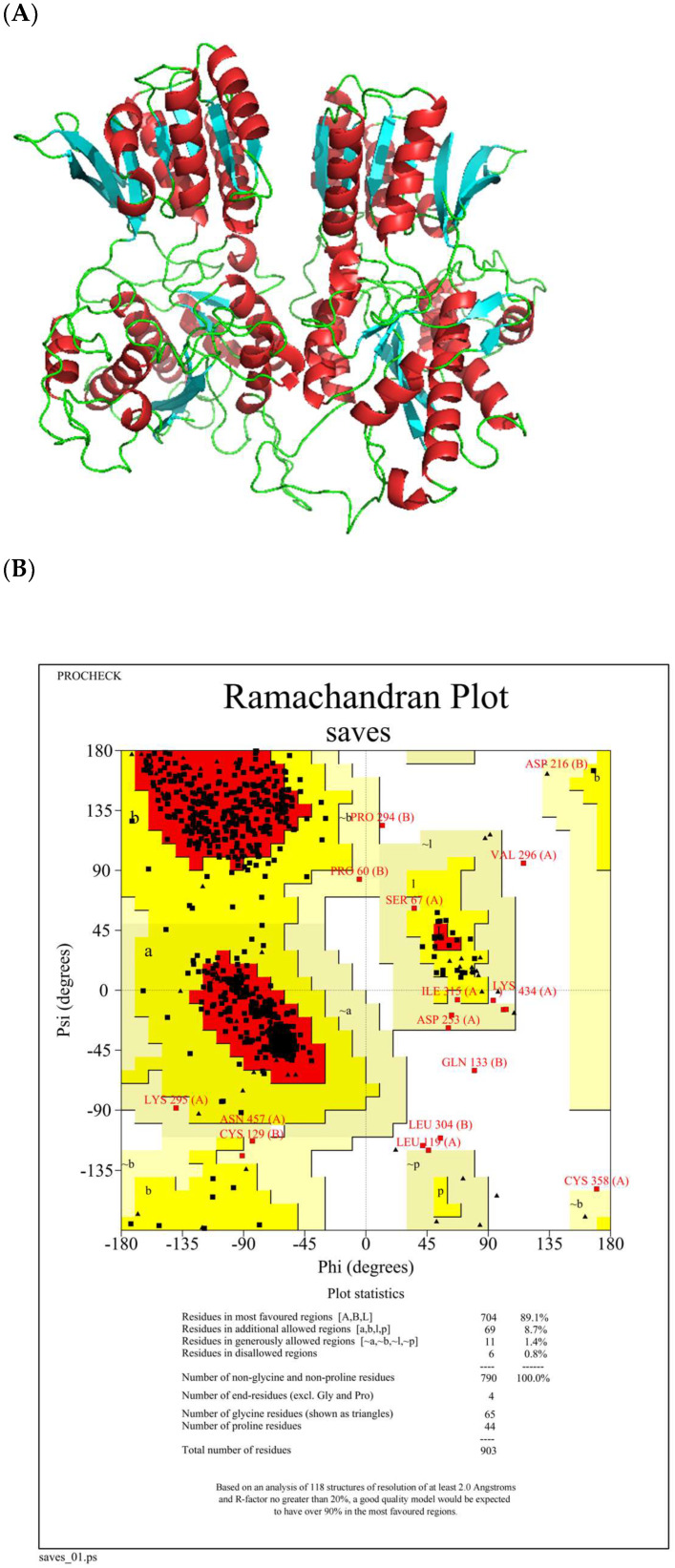
Model construction (red indicates the alpha helix, blue indicates the beta fold, and green indicates the hinge) (**A**) and Ramachandran plot (**B**) of T1R1/T1R3.

The heterodimer T1R1/T1R3, recognized as the primary receptor for umami signaling, comprises two subunits of the C-group and G protein-coupled receptors; therefore, investigating the peptide binding patterns to T1R1/T1R3 is essential for understanding the molecular basis of taste perception associated with umami peptides [20,21]. Figure 2A shows the T1R1/T1R3 receptor constructed using homology modeling, and Figure 2B shows the results of the Ramachandran-plot of this model in the SAVES 6.0 assay. The Ramachandran -plot shows that 99.5% of the amino acid residues in this homology model are located in reasonable regions (>90%), of which 87.8% of the amino acid residues are in the optimal reasonable regions; 10.1% of the amino acids are in the additional permissive regions; and 1.5% of the amino acids are in the loose and reasonable regions, which suggests that this T1R1/T1R3 model constructed using homology modeling is reasonable in terms of dihedral angle distribution and three-dimensional collision and the model can be used.

### 3.3. Taste Properties of Synthetic Peptides

Sensory evaluation and electronic tongue analysis were performed on the three peptide solutions to determine their umami taste and ability to enhance umami taste. The results are shown in Table 4 and Table 5. It was observed that the umami thresholds of YDAELS and ELELQ were 0.375 mg/mL, and 0.75 mg/mL, respectively. It is important to note that umami peptide solutions typically have a wide range of mouthfeel. YDAELS has a somewhat salty taste, TDVAHR has a somewhat astringent taste, and ELELQ also has a slightly sweet taste. The results of the electronic tongue data were consistent with the sensory evaluation. The sensory evaluation and electronic tongue analysis indicated that the umami taste of those three peptides was relatively weak, potentially confounded by the presence of salinity. This observation underlines the dual role of umami peptides, not only in manifesting a distinct taste but also in serving as potential substitutes for salt additives, thereby enhancing the health appeal and palatability of food products [22]. Furthermore, the detection of sweetness might be attributed to the shared T1R3 subunit between the sweetness and umami receptors. Contrarily, some studies have reported a bitter taste associated with synthetic peptides, specifically those resembling ELELQ [23]. The peptides TDVAHR and YDAELS examined in this study, however, did not exhibit significant bitterness. This lack of bitterness corroborates the hypothesis that umami peptides could mitigate bitter taste perceptions. Prior research has also suggested that the inherent taste profiles of synthetic peptides vary, with certain umami peptides displaying negligible intrinsic taste yet demonstrating pronounced synergistic effects when combined with monosodium glutamate (MSG) [24]. Moreover, all three peptides studied showed potential for synergistic enhancement of taste, aligning with previous findings on the interactive effects of umami peptides in flavor modulation.

The umami peptides were dissolved in 0.35% MSG solution and the umami taste was enhanced to some extent as compared to 0.35% MSG solution alone. The umami taste enhancement threshold was determined to be 0.375 mg/mL for YDAELS and 0.750 mg/mL for both TDVAHR and ELELQ. In this study, it was proven that a low concentration of umami peptides had a synergistic enhancement effect on 0.35% MSG solution. It was also proven that the excessive addition of umami peptides would also lead to a sour taste due to acidic amino acids and sodium acetate added during synthesis [25]. Further research could be conducted on the dosage of specific umami peptides and the ratio of synergistic agents. In addition to the synergistic umami-enhancing effect observed when used in conjunction with MSG, this study also revealed that umami peptides can augment the flavor of moss extract and ginger extract. This finding suggests that various flavoring compounds in food may interact synergistically with umami peptides. This could potentially lead to optimized recipes that leverage the unique properties of umami peptides to enhance the overall sensory experience [25].

Generally, the potency of umami peptides is influenced by various factors including amino acid composition, isotopic variations, and peptide chain length. Alongside their inherent structural properties, umami peptides also engage in interactions with nucleotides, organic acids, and free amino acids. Among these factors, amino acid composition emerges as the principal determinant of a peptide’s umami characteristic. For example, umami peptides usually contain acidic amino acids such as aspartate (D) and glutamate (E), while sweet amino acids such as alanine (A), serine (S), glycine (G), and threonine (T) also have an impact on umami taste. Those affecting the umami taste of peptides also include histidine (H) and valine (V). This study highlights that the peptide YDAELS includes both E and D; TDVAHR contains D; and ELELQ features E, illustrating a clear link between specific amino acids and the umami taste properties of peptides. Furthermore, it has been demonstrated that arginine (R) and lysine (K) play crucial roles in binding to ligands within umami peptides. All three peptides examined in this study—containing these amino acids—exhibited a pronounced umami flavor. Table 4 presents the umami intensity of these three synthetic peptides (at a concentration of 1 mg/mL) as assessed using an electronic tongue. The results indicated that YDAELS exhibited the strongest umami taste, while ELELQ had the weakest umami taste. There was a statistically significant variance in the umami intensity among the peptide solutions (*p* < 0.05), probably attributable to differences in amino acid composition. In summary, the solutions of the three synthetic peptides presented a generally mild taste profile, yet significant differences in umami intensity were observed.

### 3.4. Molecular Docking Reveals the Umami Presentation Mechanisms of the Three Umami Peptides

Molecular docking serves as a fundamental tool in molecular simulation, facilitating the examination of docking active sites and interaction forces between umami peptides and umami receptors. Utilizing homology modeling and molecular docking, the interactions and potential mechanisms of umami perception facilitated by these peptides can be elucidated [26,27]. In this study, a three-dimensional model of umami receptor T1R1/T1R3 molecularly docked with three umami peptides was constructed to study the umami mechanism of these three umami peptides. Molecular docking was performed between T1R1/T1R3 and three novel umami peptides using Discovery Studio 2019 Client [28,29], and the three umami peptides, YDAELS, TDVAHR, and ELELQ, could form a stable complex with the umami receptor T1R1/T1R3 [27]. The primary interactions between the three umami peptides and the T1R1/T1R3 receptor complex were predominantly governed by conventional hydrogen bonds, salt bridges, gravitational charges, carbon–hydrogen bonds, and alkyl and π-alkyl groups. A detailed analysis of these interactions at the active sites of the T1R1/T1R3 receptors is presented below.

**Figure 3:**
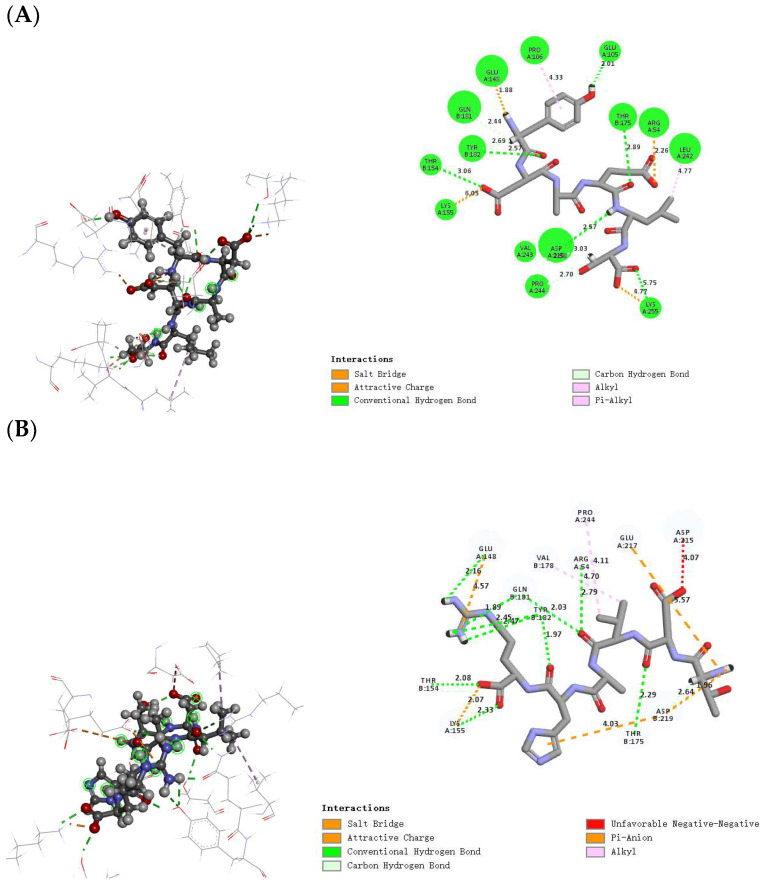

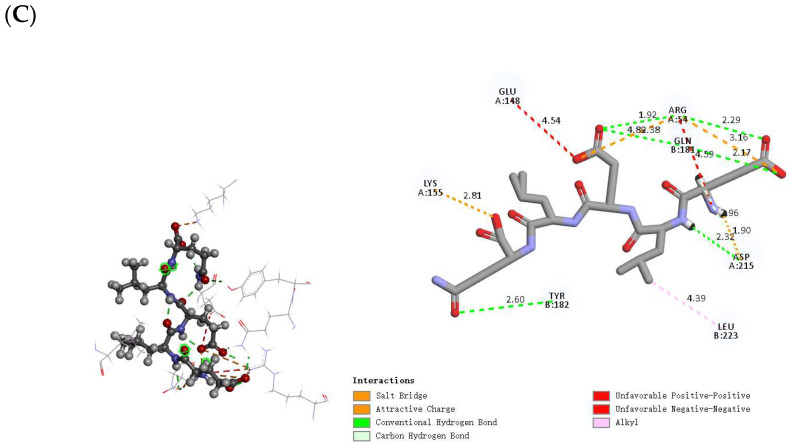
The docking (left: Gray stick structure small molecule peptide, red ball indicates oxygen element, blue ball indicates nitrogen element; Linear structures are interacting amino acid residues) and interaction (right) diagrams of (**A**) YDAELS, (**B**) TDVAHR, and (**C**) ELELQ.

As shown in Figure 3A, the ligand umami peptide YDAELS mainly interacts with the receptor T1R1/T1R3 residues through conventional hydrogen bonding, and the main sites of interaction include Glu105, Tyr182, Thr154, Asp215, Lys255, and Thr175; Gln181, Val243, and Pro244 form carbon–hydrogen bonds with the ligand umami peptide; Glu148, Lys155, Arg54, and Lys255 bind to the ligand via electrostatic interactions; and Pro106 and Leu242 bind the receptor to the ligand via hydrophobic interactions. As shown in Figure 3B, the ligand umami peptide TDVHR mainly interacts with the receptor T1R1/T1R3 residues through conventional hydrogen bonding, and the main sites of interaction include Arg54, Gln181, Tyr182, Glu148, Thr154, Lys155, and Thr175; a carbon–hydrogen bond is formed between Asp219 and the ligand umami peptide; Glu217, Glu148, Lys155, and Asp219 bind to the ligand via electrostatic interactions; and Pro244 and Val178 bind the receptor to the ligand via hydrophobic interactions. As shown in Figure 3C: the ligand umami peptide ELELQ mainly interacts with the receptor T1R1/T1R3 residues through conventional hydrogen bonding, and the main sites of interaction include Arg54, Gln181, Asp215, and Tyr182; Asp215 forms a carbon–hydrogen bond with the ligand umami peptide; Arg54, Lys155, and Asp215 bind to the ligand through electrostatic interaction; and Leu223 binds the receptor to the ligand via hydrophobic interactions. In addition, the major docking active sites of the three umami peptides, where Arg54, Gln181, Lys155, and Tyr182 were present in the docking of all three umami peptides, and these partial sites have also been previously demonstrated (Table 6) [29].

In summary, the interaction between umami peptides and the T1R1/T1R3 taste receptor was mediated by a combination of hydrogen bonding, hydrophobic interactions, and electrostatic forces. The binding sites for the three umami peptides on T1R1/T1R3 involve key residues such as Glu, Lys, Arg, and Asp. Acidic residues (Asp and Glu) contribute to the electrostatic interaction between acidic and basic amino acids, while the three hydrophobic amino acids within the umami peptides (Pro, Leu, and Val) facilitate hydrophobic interactions during molecular docking and binding to the corresponding residues on T1R1/T1R3. The interplay of electrostatic interactions between charged amino acids and hydrophobic interactions between nonpolar amino acids significantly enhances the binding affinity between the umami receptor and the umami peptides.

## 4. Conclusions

In this study, the extraction of umami peptides was carried out using ultrafiltration, and the fractions with molecular weights less than 3 kDa were selected for Nano LC-MS/MS peptide sequencing of bovine bone soup, which yielded a total of 1112 possible peptides. Toxicity prediction of the umami peptides was performed using a website, and the peptides were screened in steps, and finally, 16 peptides were screened for molecular docking. Three peptides named YDAELS, TDVAHR, and ELELQ were selected for analysis based on binding energy size. The molecular docking of the umami peptides with umami receptors T1R1/T1R3 revealed the mechanism of umami presentation, and the main interacting forces between the three umami peptides and the receptors were hydrogen bonding, electrostatic interactions, and hydrophobic interactions. Electrostatic and hydrophobic interactions were present between acidic and basic amino acids. In addition, this study explored the major docking active sites of the three umami peptides, in which Arg54, Gln181, Lys155, and Tyr182 appeared in the docking of all three umami peptides.

## Figures and Tables

**Table 1:** The top ten peptides of nano-HPLC-MS/MS and related information.

Peptide	Scan	Denovo Score	Length	m/z	z	RT	Area	Mass	ppm
SGLRLN	11,290	99	6	330.1946	2	17.45	1.29 × 10^7^	658.3762	−2.4
LATVNTN	11,146	99	7	732.3853	1	17.16	4.49 × 10^6^	731.3813	−4.6
LALSQAN	12,640	99	7	716.3901	1	19.52	2.41 × 10^6^	715.3864	−5.0
VGPS	4892	99	4	359.1912	1	7.44	1.89 × 10^6^	358.1852	−3.6
FDGDF	22,909	99	5	600.2284	1	35.30	1.37 × 10^6^	599.2227	−2.7
PAGPLG	10,612	99	6	511.2863	1	16.38	8.00 × 10^5^	510.2802	−2.3
YDPEAA	10,003	99	6	665.2755	1	15.40	6.71 × 10^5^	664.2704	−3.3
TGPAGPA	6614	99	7	570.2866	1	10.14	5.98 × 10^5^	569.2809	−2.7
LAGE	5375	99	4	389.2018	1	8.15	0	388.1958	−3.1
GPFFT	4924	99	5	568.2714	1	7.46	0	567.2693	−9.1

**Table 2:** The energy of molecular docking of peptides with T1R1/T1R3.

Fraction	Peptide	Cdocker Energy (kcal/mol)	Cdocker Interaction Energy (kcal/mol)
1	YDAELS	−129.036	−94.2538
2	TDVAHR	−129.012	−99.3804
3	ELELQ	−127.153	−82.2681
4	TDLTE	−122.007	−89.8227
5	KLLTDP	−120.47	−82.3731
6	TEELA	−114.989	−85.419
7	FDGDF	−113.031	−79.4462
8	YDPEAA	−108.955	−87.4857
9	TPVSDR	−108.792	−101.904
10	LAFTD	−108.216	−79.2393
11	TVEAS	−104.411	−77.2828
12	LSEL	−100.613	−71.9283
13	EVLQ	−98.5986	−71.1456
14	FVMD	−91.8658	−81.3383
15	VGPEPQ	−91.2323	−81.283
16	LAGE	−88.396	−56.0074

**Table 3:** MS/MS result of the three peptides.

Peptide	YDAELS	TDVAHR	ELELQ
Scan	13138	3813	16485
Denovo score	98	98	95
Length	6	6	5
m/z	697.3005	349.6811	631.3275
z	1	2	1
RT	20.19	5.76	25.47
Area	3.89 × 10^5^	1.30 × 10^5^	1.04 × 10^6^
Mass	696.2966	697.3507	630.3224
ppm	−4.9	−4.4	−3.6

**Table 4:** Electronic tongue analysis of the three synthetic peptides.

	Sourness	Bitterness	Astringency	Aftertaste-B	Aftertaste-A	Umami	Richness	Saltiness
YDAELS	−44.45 ± 2.28 ^c^	−0.67 ± 0.27 ^b^	−16.85 ± 1.58 ^b^	2.65 ± 0.32 ^a^	−0.69 ± 0.07 ^a^	12.72 ± 0.80 ^a^	5.21 ± 0.26 ^a^	29.98 ± 1.67 ^a^
TDVAHR	−25.22 ± 1.38 ^b^	−1.49 ± 0.44 ^b^	−15.87 ± 0.89 ^b^	1.62 ± 0.24 ^b^	−1.68 ± 0.27 ^a^	7.02 ± 0.24 ^b^	5.09 ± 0.49 ^a^	16.18 ± 0.78 ^b^
ELELQ	−3.23 ± 0.50 ^a^	10.36 ± 0.81 ^a^	12.93 ± 1.33 ^a^	2.19 ± 0.46 ^a^	−0.54 ± 0.11 ^a^	1.47 ± 0.26 ^c^	4.81 ± 0.69 ^a^	3.38 ± 0.29 ^c^

Data are represented as mean ± SD. a–c indicated the significance difference (*p* < 0.05).

**Table 5:** The umami threshold of synthetic peptides and the umami threshold of enhancement.

Peptide	Basic Taste	Umami Threshold Value (mg/mL)	Umami-Enhancing Threshold Value (mg/mL)
YDAELS	Salty, weak umami, weak astringent, weak bitter	0.375 ± 0.05 ^b^	0.375 ± 0.05 ^b^
TDVAHR	Astringent, weak sweet	0.75 ± 0.05 ^a^	0.75 ± 0.10 ^a^
ELELQ	Sweet, weak bitter, weak salty	0.375 ± 0.10 ^b^	0.75 ± 0.05 ^a^

Data are represented as mean ± SD. a,b indicated the significance difference (*p* < 0.05).

**Table 6:** Amino acid residues that interact with T1R1/T1R3 of three umami peptides.

	YDAELS	TDVAHR	ELELQ
Arg54	+	+	+
Asp215	+		+
Asp219		+	
Gln181	+	+	+
Glu105	+		
Glu148	+	+	
Glu217	+		
Leu223			+
Leu242	+		
Lys155	+	+	+
Lys255	+		
Pro106	+		
Pro244	+	+	
Thr154	+	+	
Thr175	+	+	
Tyr182	+	+	+
Val178		+	
Val243	+		

## Data Availability

The data presented in this study are available from the corresponding author upon request. The data are not publicly available due to privacy restrictions.
